# Efficacy of EPS Gel Coating and Lactic Acid Bacteria in Preserving Strawberry Postharvest Quality

**DOI:** 10.3390/gels12040341

**Published:** 2026-04-19

**Authors:** Dahiana Erazo Anacona, Daniela Neira Garzón, Anna María Polanía Rivera, Cristina Ramírez-Toro, German Bolívar Escobar

**Affiliations:** 1 Faculty of Engineering, School of Food Engineering, Universidad del Valle (University of Valle), Tuluá 763021, Colombia; dahiana.erazo@correounivalle.edu.co (D.E.A.); daniela.neira@correounivalle.edu.co (D.N.G.); 2 Faculty of Engineering, School of Food Engineering, Universidad del Valle (University of Valle), Cali 760031, Colombia; cristina.ramirez@correounivalle.edu.co; 3 Department of Biology, Faculty of Natural and Exact Sciences, Universidad del Valle (University of Valle), Cali 760031, Colombia; german.bolivar@correounivalle.edu.co

**Keywords:** strawberry, edible coating, exopolysaccharide, post-harvest loss, shelf-life

## Abstract

Post-harvest economic losses in the strawberry industry are predominantly driven by disease caused by food-contaminating fungi and the loss of physicochemical quality. The gel-like matrix of the edible coating (EC) serves as a carrier for delivering lactic acid bacteria (LAB). This structured network, characterized by its excellent adhesion and film-forming stability, maintains fruit integrity and delivers antifungal properties to control pathogens. This study tested an exopolysaccharide coating with *L. plantarum* A6 to assess its impact on strawberry shelf life and quality stored at 4 °C and 30 °C for eight days. Through the analysis of physicochemical properties, it was possible to observe that weight loss increased during storage time in all treatments except for strawberries coated (CF) at 4 °C, with 2.43 ± 0.2%. Regarding firmness, after 8 days of storage the greatest difference occurred in the fruit uncoated (UF) exposed to 30 °C decreased 30.93%, whereas the CF group showed a reduction of 2.04%, showing a significant difference between these treatments (*p* < 0.05). However, the CF at 4 °C had a value of 3.98 ± 0.3 N after eight days of storage, which is close to that of fresh fruit, indicating the effectiveness of the coating. In terms of microbiological results, the mesophilic and mold counts were lower in the treatment at 4 °C with coating (3.6 log CFU/g and 4.48 log CFU/g) than in the treatment stored at 30 °C (5.78 log CFU/g and 6.04 log CFU/g). The shelf-life estimate determined that CF stored at 4 °C could be preserved for 15 days and those stored at 30 °C for 6 days. Finally, sensory evaluation determined that CF stored at 4 °C were well-accepted in terms of attributes such as taste, sweetness, firmness, and flavor. These findings underscore the effectiveness of coating, not only in improving the storage quality of strawberries, but also in ensuring their sensory acceptance.

## 1. Introduction

The strawberry (*Fragaria × ananassa*) is a fruit of high nutritional and sensory value, widely appreciated for its flavor, aroma, and content of bioactive compounds. It provides benefits such as low-calorie content and a high contribution of antioxidants, primarily vitamin C, which plays a role in the formation of bones, teeth, and red blood cells [1]. However, its highly perishable nature represents a significant challenge for its commercialization and postharvest preservation [2]. According to Mordor Intelligence (2024), the global strawberry market reached a size of USD 14.7 billion in 2024 and is expected to grow to USD 18 billion by 2030, with a compound annual growth rate of 4.1%. The main producing countries are China with 40.2%, the United States with 11%, Egypt with 7%, and Turkey with 6.5% [3]. In Colombia, strawberries are cultivated year-round, with notable varieties including Camarrosa, Albion, Camino Real, Monterrey, San Andreas, Portola, Ventana, and Palomar [4]. The area planted with strawberries in 2024 was 3658 hectares, with the main producing departments being Cundinamarca, Antioquia, and Cauca [5].

In Valle del Cauca, municipalities such as Tuluá have demonstrated significant potential in agricultural production, benefiting from government programs like ‘Valle Agro’, which has supported over 100 rural families with agricultural inputs and technical assistance to improve their crops. Additionally, initiatives such as “Contract Farming” have facilitated business opportunities for local producers, promoting fruit commercialization and strengthening the agricultural sector in the municipality [6]. These efforts highlight the growth and importance of strawberries in the Colombian market, as well as the need for strategies to enhance their preservation and quality during storage.

However, the commercialization of fresh strawberries faces significant challenges due to postharvest losses, attributable to factors such as harvesting at an inappropriate stage of the ripening process, excessive exposure to rain, drought, or extreme temperatures, physical damage that contributes to reducing the value of the product (estimated at between 15% and 50% of production in developing countries) [7], and the growth of pathogenic microorganisms such as *Botrytis cinerea*, which causes gray mold, *Rhizopus stolonifer* and *Mucor* spp. which cause soft rot, *Colletotrichum* spp. which causes anthracnose disease, and finally those causing other postharvest rots of lesser incidence such as *Penicillium expansum*, *Aspergillus* spp. [8]. These losses affect the availability of the product on the market, the profitability of the producers, and the satisfaction of consumers.

In this context, the use of edible coatings (ECs) has appeared as a viable strategy to extend the shelf life of strawberries. These ECs are an advanced post-harvest solution that acts as a semi-permeable membrane, efficiently regulating fruit respiration and transpiration. In addition to these barrier properties, they help preserve visual quality, reduce physical damage, and microbial contamination [9,10]. Recent research has shown that the application of biopolymer-based coatings, such as sodium alginate, exopolysaccharides or chitosan can significantly improve fruit preservation because it acts as a barrier against oxygen and CO_2_, creating a cover against water vapor, which prevents dehydration and maintains the firmness of the fruit [11,12,13]. For instance, in the research conducted by Zhou et al. [14], a coating combining mulberry anthocyanin extract from *Morus nigra* fruits and carboxymethyl chitosan was developed to extend the postharvest life of strawberries. The results showed a significant reduction in weight loss, preserved firmness, and decreased malondialdehyde accumulation compared to other evaluated treatments. Similarly, the study by Pié-Amill et al. [15] demonstrated the effectiveness of an edible coating with the addition of carvacrol and citral. When applied to strawberries via immersion and subsequently frozen, it was observed that all bacteria were reduced to less than 0.7 log CFU/strawberry by the eighth week, and the infectivity of Murine Norovirus (MNV-1) showed a reduction of nearly 2 log units.

On the other hand, lactic acid bacteria (LAB) have been the subject of research due to their capacity to inhibit the growth of phytopathogenic fungi, offering a natural alternative for fruit preservation. Studies have shown that some LAB strains produce antimicrobial compounds effective against fungal pathogens [16]. Additional effects reported for LAB application include enhanced water vapor permeability [17], as well as potential alterations to the color and light transmission of the edible films [18]. Research such as that conducted by Álvarez et al. [13] shows that the incorporation of *L. plantarum* A6 in an edible coating applied to tomatoes successfully inhibited the growth of *Fusarium sp*. and *Rhizopus stolonifer*. Similarly, Menéndez-Cañamares et al. [19] incorporated *Bacillus subtilis* SB8 into a coating applied to strawberries, achieving a reduction in infection by *B. cinerea*. In another study conducted by Doukaki et al. [20], who applied edible coatings enriched with *Lactiplantibacillus pentosus* L33 and *Lactiplantibacillus plantarum* L125 to feta cheese, demonstrated a shelf-life extension of 13 days compared to control samples, as well as a mild antimicrobial effect against *L. monocytogenes.* Finally, the study realized by Li et al. [2] mentions that the use of sodium alginate coatings enriched with *L. rhamnosus* on strawberries serves a threefold purpose: it physically protects the fruit, provides antibacterial properties, and gives the product potential probiotic benefits for consumers. Consequently, this study focuses on evaluating how an optimized edible coating formulation (containing lactic acid bacteria and exopolysaccharide) affects the preservation and shelf life of strawberries under two different temperature conditions (4 °C and 30 °C).

## 2. Results and Discussion

### 2.1. Effect of Coating on Strawberry Physicochemical Quality

#### 2.1.1. Weight Loss

**Figure 1:**
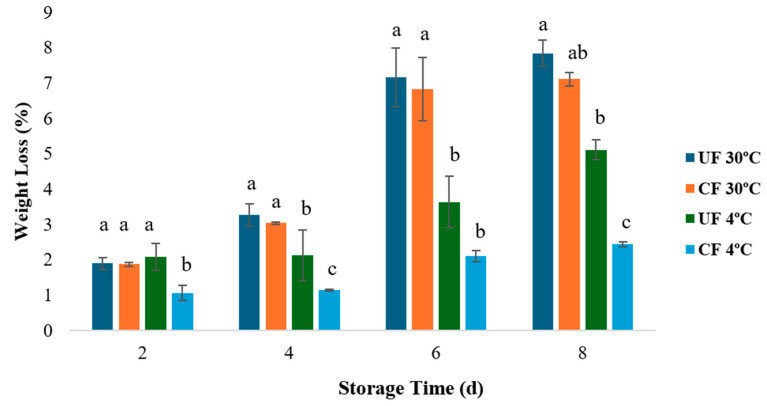
Changes in weight loss in coated (CF) and uncoated (UF) strawberries stored under refrigerated conditions (4 °C) and at room temperature (30 °C). Data are presented as the mean ± standard deviation (n = 3). Different letters indicate significant differences between treatments based on the significance test (*p* < 0.05).

A significant increase in weight loss was observed in all strawberry samples as the storage period progressed. This phenomenon is primarily attributed to the fruit’s respiration and transpiration processes, as illustrated in Figure 1. According to the statistical analysis performed, there were no statistically significant differences between the treatments at room temperature (30 °C), as the weight loss at the end of storage was 7.84% (UF) and 7.11%, respectively. However, there were statistically significant differences (*p* < 0.05) between the treatments at refrigeration temperature (4 °C), with a lower weight loss in coated strawberries (2.43 ± 0.2%) compared to (5.1 ± 0.3%) in uncoated samples, representing a difference of 2.7%. Considering the mentioned by Kessler et al. [21], the commercial shelf life of strawberries is compromised when weight loss exceeds the 6% threshold, as visual quality and turgidity decrease significantly. The treatments that fell below this value were the samples stored under refrigeration, with the coated strawberries falling well below this threshold.

These results indicate that, under environmental conditions, the high vapor pressure gradient and increased respiration rate favor water loss from the fruit, partially overcoming the protective capacity of the composite coating. Although the inclusion of oleic acid provides some resistance to moisture transfer, the polysaccharide matrix (alginate and exopolysaccharide) remains hydrophilic and, therefore, sensitive to water vapor. This is consistent with the literature, which reports that polysaccharide-based films lose effectiveness as a transpiration barrier at high temperatures and low relative humidity [22,23,24].

Furthermore, in warm and dry environments, the rapid dehydration of the pericarp can induce microcracks or loss of film adhesion, which creates preferential routes for moisture to escape and further limits its protective effect. Epidermal microcracking as a pathway for postharvest water loss has been documented in fruit trees and is intensified under conditions of superficial water stress [25,26].

The samples were refrigerated at 4 °C, and the coating showed a consistent protective effect. This behavior is explained by the fruit’s postharvest physiology, since the low temperature reduces both respiration and transpiration. This is aligned with described in the literature, which reports clear decreases in mass loss in strawberries when combining polysaccharide-lipid coatings with a cold chain [24,26]. Specifically, studies such as those by [27,28], using alginate/CMC-based coatings plus a lipid phase and/or bioactive agents, report 30–40% reductions in final weight loss during storage at 4–5 °C, which is consistent with the results obtained in this study. At a materials level, recent strategies to reduce the water vapor permeability (WVP) of polysaccharide films (incorporating lipids/emulsions, reinforcing with nano/micro-hydrophobic phases, or using bilayers) explain why, in cold conditions where the vapor gradient is lower and the film remains intact, the barrier works better and translates into less accumulated dehydration [22,23]. This phenomenon is attributed to the barrier properties of the coating, which effectively reduce moisture diffusion through the strawberry’s stomata. Furthermore, the incorporation of lactic acid bacteria into edible coatings likely exerted antimicrobial effects that inhibited the growth and metabolic activity of spoilage microorganisms at the food surface, thereby contributing to reduced matrix degradation and improved retention of moisture and nutrients [29]. Similarly, the study conducted by Li et al. [2] on alginate films functionalized with lactic acid bacteria showed trends consistent with the results obtained in the present study.

#### 2.1.2. Color

The results obtained for the L* coordinate (lightness, associated with the fruit’s brightness and perception of freshness) showed a decrease in all treatments throughout storage, reflecting the progressive browning characteristic of postharvest strawberries (Table 1). Under control ambient conditions, lightness decreased from 38.70 ± 3.82 to 8.92 ± 1.13, showing the most drastic decline. For the coated fruit under ambient conditions, the values decreased from 39.40 ± 3.58 to 13.88 ± 0.12, exhibiting a similar browning pattern, albeit with slightly higher final values than the control. In the control refrigeration treatment, the values changed from 37.73 ± 0.51 to 11.99 ± 1.76, indicating a considerable loss. Finally, the combination of refrigeration and coating demonstrated the best performance, starting at 39.77 ± 3.52 and ending at 21.22 ± 2.77, confirming that this treatment most effectively preserved lightness. According to the mean comparison analysis using Tukey’s test, the brightest sample was the refrigerated and coated one, showing a statistically significant difference (*p* < 0.05). This tendency toward greater opacity in the 30UF treatment is a phenomenon probably associated with microbial growth on the surface of the fruit starting on day four. In this regard, the treatment that showed greater brightness on day 8 was the one stored under refrigeration and coated. This behavior correlates with that reported by Zhou et al. [30], who observed a downward trend in strawberry clarity in all treatments evaluated during a 15-day storage period.

These trends are similar with studies realized by [31,32], who reported that the decrease in L* (lightness) in strawberries during storage is associated with anthocyanin degradation and oxidative reactions. On the other hand, the use of edible coatings (chitosan, pectin, nanocellulose) helps retard this decline by limiting oxygen exposure and reducing transpiration, thereby preserving the fruit’s color and fresh appearance [32,33]. González-Cuello et al. [32], it was observed that strawberries coated with a multilayer biopolymer maintained higher L* values compared to controls which coincides with the results obtained in this research. Similarly, recent research on coatings enriched with essential oils and chitosan nanoparticles has demonstrated a significant reduction in total color change (ΔE) and better preservation of L* compared to uncoated fruit [34,35].

Regarding coordinate a*, the trend can be seen in Table 1. The a* coordinate, associated with red color intensity, it was observed that fruits stored at room temperature exhibited an initial increase in both treatments, followed by a decrease. This behavior aligns with the typical evolution of strawberries as ripening progresses and oxidative processes begin. However, the reduction in a* was more abrupt in the control group, whereas in the coated strawberries the decline was more attenuated. This suggests that the edible coating delayed the degradation of anthocyanins and contributed to maintaining greater stability of the red color towards the end of the storage period. This protective effect of coatings on pigments has been reported in studies with coatings based on chitosan nanoparticles, which better preserve the firmness and pigments in refrigerated strawberries [36].

However, refrigeration remains the factor that most significantly influences the preservation of the red color, as both the coated fruits and the controls stored at low temperature maintained higher and more stable a* values. This confirms that the storage temperature is decisive for preserving the fruit’s visual appearance, which can be attributed to the fact that storage at 4 °C favors the activation of antioxidant systems such as peroxidase (POD) and helps to better maintain visual quality, even though higher levels of anthocyanins accumulate at room temperature [37]. This behavior is supported by the statistical analysis; by day 8, no statistically significant differences were observed between the refrigerated treatments (coated strawberries: 38.31 ± 0.38 and uncoated strawberries: 37.33 ± 0.45). However, there were statistically significant differences (*p* < 0.05) between the treatments at room temperature (coated strawberries: 26.57 ± 0.58 and uncoated strawberries: 21.21 ± 0.32).

For the b* coordinate, which reflects the yellow-blue tone, a progressive decrease was observed in all treatments, reflecting the loss of pigments and phenolic compounds characteristic of the fruit during storage. Under ambient conditions, both the control and the coated samples exhibited a more pronounced drop in b*, evidencing an accelerated progression of ripening and visual deterioration. In contrast, under refrigeration, although a decrease was also recorded, it was more gradual and with less variability, confirming that low temperature delays pigment degradation. The effect of the coating was more evident at 4 °C, where it helped to attenuate the loss of tone by limiting dehydration and oxidation, acting as a protective physical barrier. These results align with recent reports, which demonstrate that edible coatings can slow down color and quality deterioration in strawberries under different storage conditions [38,39]. Statistical analysis showed no statistically significant differences between the uncoated samples stored at 4 °C and 30 °C. However, significant differences were found among the coated treatments at different temperatures. The b* coordinate was higher in the refrigerated coated sample (29.35 ± 2.02), as fruits under these conditions better preserve their original optical properties.

Regarding the hue angle (Actg (b*/a*), a progressive decrease was observed in all treatments, reflecting a shift from bright tones towards more reddish hues as storage progressed. Under ambient conditions, the control changed from 49.85° to 40.23°, while the coated sample decreased from 53.43° to 40.69°, showing a less pronounced decline. For this parameter, there were statistically significant differences between temperatures (4 and 30 °C) (*p* < 0.05). However, hue values were similar between treatments at 4 °C and 30 °C, with a lower loss of hue observed in the refrigerated samples (27.43 ± 1.39 for UF and 26.77 ± 1.92 for CF). These results suggest that the edible coating contributed to partially delaying the loss of hue, especially in combination with refrigeration. Similar trends have been reported in the literature with other coating formulations. For example, Ref. [36] demonstrated that films based on chitosan and glycine betaine preserve the chromatic appearance of strawberries during cold storage, while Elabd and Zhale [33] highlight that biopolymers and nanomaterials act as barriers against oxidation and pigment degradation. Both the experimental results and previous evidence confirm that the use of edible coatings, regardless of the specific formulation, constitutes an effective strategy for prolonging the visual quality of fresh strawberries.

#### 2.1.3. Firmness

**Figure 2:**
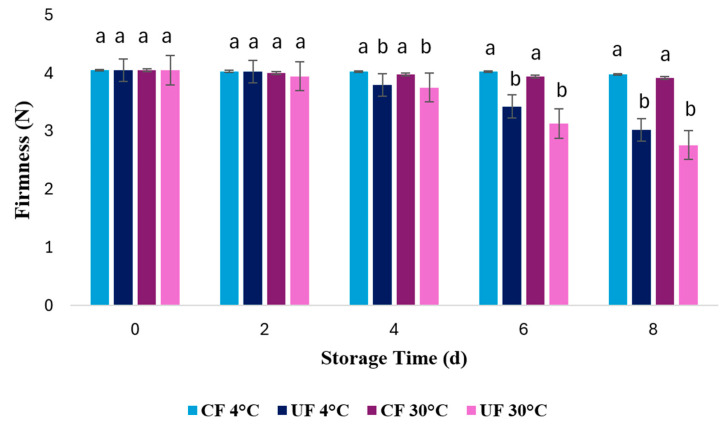
Loss of firmness of coated (CF) and uncoated strawberry fruit (UF) during storage at room temperature (30 °C) and under refrigeration (4 °C). Values are expressed as mean, and error bars denote standard deviation (n = 3). Values with different superscript letters (a, b) on the same bar indicate significant differences (*p* < 0.05).

Firmness is an important indicator of strawberry texture and a relevant quality parameter at the time of purchase. As shown in Figure 2, the firmness of the strawberries decreased significantly during storage in every treatment group. The greatest change in firmness was found in the UF treatment at 30 °C, as on day 8 this parameter decreased by 30.93% compared to the samples coated at the same temperature, which decreased by 2.04%, reflecting the effectiveness of the coating. This loss of mechanical integrity is expected, as fruit ripening involves the hydrolysis of the cell wall matrix (cellulose, hemicellulose, and pectin). Specific enzymes like polygalacturonase, cellulase, and pectinmethylesterase drive this breakdown, resulting in the observed reduction in firmness [40].

Particularly on day 8 of storage, a statistically significant difference (*p* < 0.05) was observed between the CF and UF samples, with the firmness of coated strawberries being 36.3% greater than that uncoated. Furthermore, the coating effectively maintained fruit hardness through day 8 of storage. The lowest firmness values were observed in uncoated fruit stored at 30 °C (2.763 N) and uncoated fruits stored at 4 °C (3.025 N) on day 8. Similar results were reported by [41], who observed a decrease in firmness for all treatments evaluated, with the best treatment being coating with hyaluronic acid nanocomposites. Similarly, Ref. [32] reported a 27.78% reduction in firmness in the control group, while coated strawberries only decreased by 15.17%.

The results obtained are consistent with those reported by De Bruno [41], who analyzed the impact of edible coatings enriched with natural antioxidants on the physicochemical quality of strawberries stored under refrigeration for up to 14 days. It was determined that the structural integrity of the fruit was maintained through the combined effect of the coating, the use of gum arabic, and the addition of natural extracts, which acted in concert. Specifically observed that fruit lacking the edible coating exhibited a significantly faster decrease in firmness than fruit treated with the extract-enriched coatings during the storage period. Likewise, it aligns with the findings reported by Zebua et al. [39], who evaluated the effect of edible coatings with different concentrations of lemon peel extract on the physicochemical and sensory quality of strawberries stored at 4 °C. They reported that firmness decreased in all treatments, but the coated strawberries showed a higher firmness value than the control and were able to significantly maintain the fruit’s hardness level until day 8.

Likewise, in the study by Zahra [42], developed an edible coating (EC) formulated with xyloglucan (XG) and a bioactive compound—specifically, an extract from Borassus flabellifer seed coat (BFE). The study then investigated how this coating affected strawberry quality during an 8-day storage period under ambient conditions. The authors reported that coated strawberries (XG and XG/BFE treatments) exhibited a significantly slower rate of firmness loss compared to the control within the first 5 days of storage, with the control sample showing the lowest firmness (2.22 N). This highlights the effectiveness of the XG/BFE coating treatments in delaying tissue softening and maintaining higher firmness. According to García-Betanzos [43], this behavior is attributed to the barrier property of the coatings, which create a modified atmosphere around the surface of the fruit, reducing the exchange of metabolic gases (oxygen and carbon dioxide) and limiting the activity of the enzymes responsible for softening.

#### 2.1.4. Total Soluble Solids, pH and Titratable Acidity

The pH of strawberries is crucial for determining their flavor profile. As shown in Table 2, the initial pH of the strawberries (averaged across all treatment groups on day 0) was 3.42 ± 0.05. The uncoated strawberries at 30 °C showed a pH increase to 3.60 ± 0.02, while the refrigerated ones had a pH of 3.55 ± 0.02 on day 8 of storage. Meanwhile, coated strawberries stored at 4 °C showed a smaller increase in pH on day 8 (3.52 ± 0.01). This increase in pH is likely a consequence of the respiration process. During respiration, organic acids are consumed as a substrate in enzymatic reactions, which convert these acids into sugars, thereby causing the pH to rise [44]. According to [45], citric acid, which is the main organic acid in ripe strawberries regardless of the treatment, is the cause of the pH decrease that occurs during storage. The observed trend as well as the values are consistent with what was reported by [46,47,48].

Titratable acidity (TA) is also a parameter that directly influences the flavor of strawberries [49], reflecting the content of organic acid present in the fruit, which decreases over time due to metabolic changes during fruit ripening and its use in respiration [50]. As shown in Table 2, the coating maintained a constant TA throughout storage at both temperatures, suggesting a delay in the utilization of organic acid. These results are consistent with what was reported by [42], who developed an edible coating based on xyloglucan in combination with a Borassus flabellifer seed coat extract to extend the postharvest shelf life of strawberries for 8 days of storage. Sharafi et al. [48] also reported this trend in titratable acidity in their research on edible coatings on strawberries based on flaxseed mucilage enriched with postbiotics.

Total Soluble Solids (TSS) are a key parameter in assessing fruit quality. Through storage and ripening, TSS levels fluctuate as a result of polysaccharide degradation, tissue dehydration, and the resulting concentration of sugars in the juice [51]. Starting at 9.26 ± 0.05 °Brix, the total soluble solids (TSS) in the treated samples decreased progressively from day 2 through the end of the study period (day 8). This trend is attributed to the use of sugars as a substrate for cellular respiration during ripening. Since the strawberry is a non-climacteric fruit, its metabolic activity and carbohydrate degradation continue steadily after harvest [41]. However, greater stability was observed in the coated and refrigerated samples, which could be attributed to the fact that the coatings act as a gas barrier, limiting the respiration of the fruit by suppressing oxygen absorption [42].

### 2.2. Oxygen Consumption, Respiration Rate and Ethylene Production

**Figure 3:**
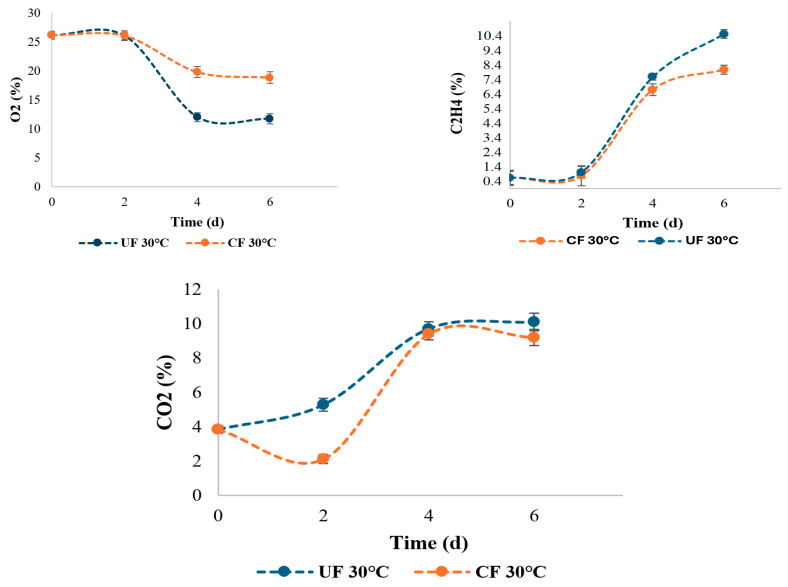
Oxygen consumption, carbon dioxide production and ethylene production in the samples at room temperature (30 °C) for each of the treatments.

In the current study, the EC decreased the respiration rate of strawberries (Figure 3). A more marked decrease in oxygen (O_2_) and an increase in carbon dioxide (CO_2_) were observed in coated strawberries stored at room temperature compared to uncoated samples. This behavior indicates that the edible coating acted as a semipermeable barrier to gas exchange, reducing the availability of O_2_ and retaining the CO_2_ generated by respiration. This effect has been reported in strawberries, where coatings reduce oxygen permeability and help maintain postharvest quality [41].

These results are consistent with studies where edible coatings act as a semipermeable barrier that modifies the internal atmosphere, reducing the respiration rate and extending the shelf life of the fruit [52,53]. In addition, it has been reported that the reduction of oxygen inhibits the activity of ACC oxidase, while a slight increase in CO_2_ can suppress both ACC synthase and oxidase, thus delaying ethylene biosynthesis and senescence [54]. In other studies, it is highlighted that coatings also minimize gas exchange and regulate the balance of CO_2_ and O_2_, delaying metabolic processes that lead to excessive ripening or deterioration [53,55].

Regarding ethylene production, strawberries in a coated environment showed lower values compared to uncoated samples. This result is related to the function of the edible coating as a physical barrier to gas exchange, which reduces the respiration rate and, consequently, ethylene synthesis. Ethylene is a plant hormone that, although produced in small quantities in non-climacteric fruits like strawberries, is associated with softening, loss of firmness, and the acceleration of senescence processes. Therefore, a lower accumulation of ethylene in the coated fruits indicates a delay in the physiological changes that lead to the deterioration of postharvest quality. This behavior has been reported in the literature, where the application of alginate or polysaccharide coatings reduces ethylene production and extends the shelf life of strawberries and other berries [41].

**Figure 4:**
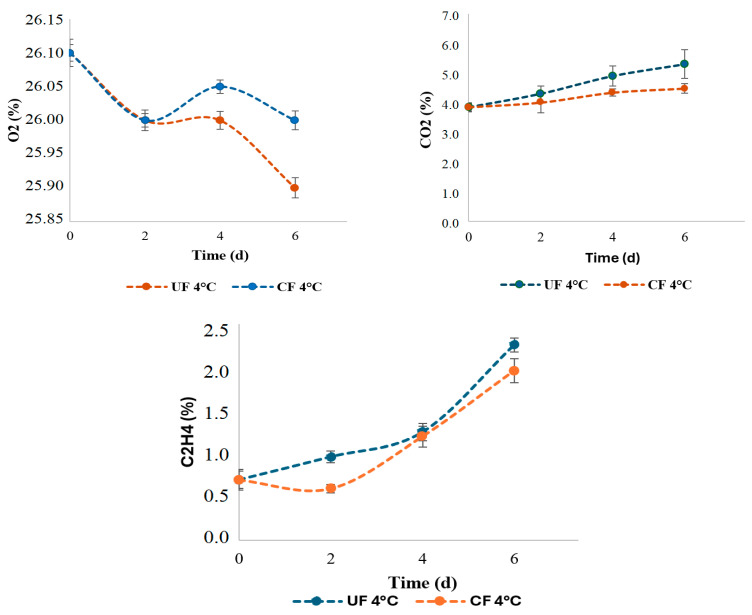
Oxygen consumption, carbon dioxide production and ethylene production in the samples at refrigerate temperature (4 °C) for each of the treatments.

During refrigeration (Figure 4), the oxygen (O_2_) content in both treatments remained relatively stable, close to 26%, which confirms that low temperature significantly reduces strawberry respiration. However, the curve for the coated samples (UF 4 °C) shows a slight additional decrease in O_2_ compared to the control samples (CF 4 °C), indicating that the coating acted as a partial barrier to gas exchange, reducing oxygen consumption and, therefore, metabolic activity. This effect is consistent with previous reports where edible polysaccharide or pectin coatings decrease O_2_ permeability and delay processes associated with respiratory metabolism [41].

Regarding carbon dioxide (CO_2_), the control strawberries accumulated higher levels over time, reaching almost 6%, while the coated ones remained around 4.5%. This demonstrates that the coating not only limited oxygen consumption but also moderated CO_2_ production, reflecting a slower respiratory metabolism. This behavior is favorable, as moderate CO_2_ levels help reduce the incidence of *Botrytis cinerea* and delay senescence, while excessive accumulations can induce fermentation and undesirable flavors [56,57].

In the present study, strawberries stored under refrigeration with a coating showed lower ethylene concentrations compared to the control treatment. This reduction is consistent with findings in the literature, where various authors have demonstrated that edible coatings act as a semi-permeable barrier that restricts gas exchange, lowers the respiration rate, and consequently reduces ethylene synthesis. A comparative study on strawberries coated with carboxymethyl cellulose, pectin, or gums showed better preservation of phenolic compounds, anthocyanins, and less physical deterioration compared to the control when the fruits were stored at 4 °C [58]. Although these compounds are not directly responsible for ethylene production, they are considered relevant because their degradation is accelerated by metabolic processes linked to respiration and ethylene. Therefore, their preservation indicates a delay in ripening. Similarly, research with chitosan-enriched coatings has shown that they help maintain firmness, reduce weight loss, and preserve the necessary soluble solids, suggesting that they work to decrease internal gases that promote degradation [59].

### 2.3. Microbiological Tests

**Figure 5:**
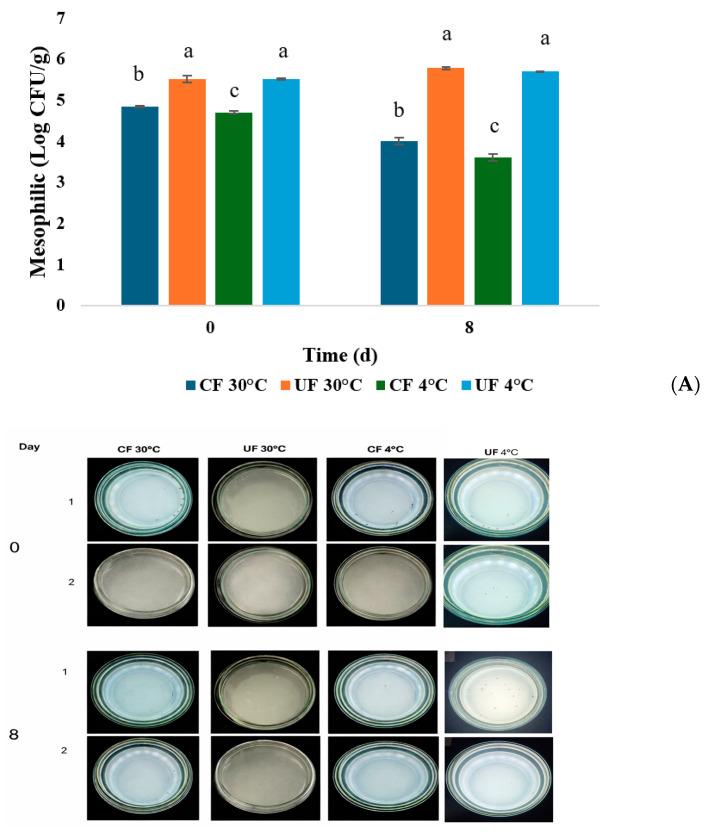

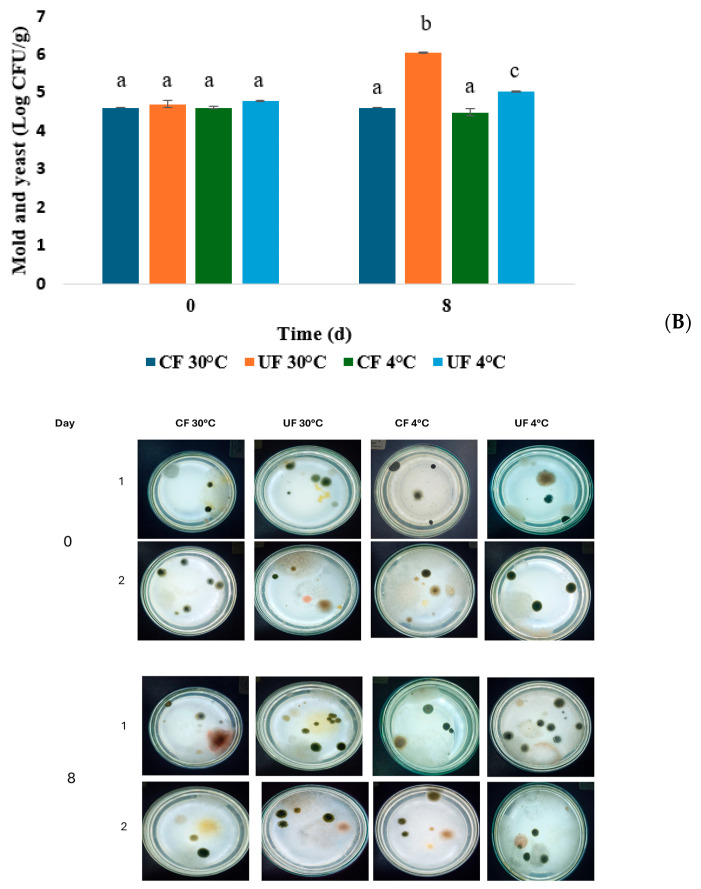
Assessment of microbial load in coated (CF) and uncoated (UF) strawberries stored under two temperature conditions: refrigerated (4 °C) and ambient (30 °C). The numbers represent the counts of (**A**) mesophiles and (**B**) yeasts and molds. Values are expressed as the mean, and error bars indicate the standard deviation (n = 3). Values with different letters (a–c) on the same bar indicate significant differences (*p* < 0.05).

In the coated strawberries, total mesophile counts revealed that the only Colony Forming Units (CFU) present were the lactic acid bacteria (LAB) incorporated into the coating (Figure 5). This was confirmed because only one type of colony was observed on the plates, which was identified as *L. plantarum* based on its morphological characteristics. While the number of viable cells decreased over time, the bacterial population remained above 1 × 10^4^ CFU/g at both temperatures. This is considered a promising result that demonstrates the viability of the bacteria in the coating solution. The microbiological values obtained are within the acceptable limits established by Colombian regulations (Resolution 1407 of 2022), which includes a maximum aerobic plate count of 3 × 10^3^ CFU/mL at the end of the shelf life for various fresh-cut fruits and vegetables.

The main reason that bacteria survive better in food is, according to some research, due to the specific composition of the formulations of the edible coatings (EC). One of the studies that reported something similar was that of [60], who mention that the growth of *L. plantarum* is largely due to the inclusion of oleic acid in the coated grapes because it acts as a surfactant, improving the coating’s adherence to the fruit, which in turn favors the adherence of the microorganism. This same behavior was observed by Álvarez et al. [13] applying LAB coatings to cherry tomatoes. Statistical analysis showed that there were no statistically significant differences in the uncoated samples on day 0, but there were differences in the coated treatments. On day 8, there was less growth in the coated and refrigerated samples, with significant differences (*p* < 0.05) compared to the uncoated samples, highlighting the synergistic effect of the coating with the refrigeration temperature.

Mold and yeast counts were greater in the uncoated samples stored at room temperature (30 °C) than in those stored under refrigeration (Figure 5). This count was similar to that reported by De Bruno et al. [41] who applied coatings enriched with bergamot essential oil to strawberries. During the storage period, the researchers observed increases in all analyzed microbiological parameters, especially at 14 days of storage. The samples with the greatest microbiological deterioration were those without coatings. These values demonstrated that the application of edible coatings is useful for improving and prolonging the quality of strawberries. The results obtained in this study were similar to those reported by [61], who, specifically for yeasts and molds, reported populations of 2.81 ± 0.12 log_10_ CFU/g and 3.68 ± 0.07 log_10_ CFU/g for the refrigerated coated strawberry and the control sample, respectively. In this research, the value was 4.60 ± 0.02 and 4.48 ± 0.07 log_10_ CFU/g, respectively. Regarding the microbiological counts for molds and yeasts, the results also met the limits established by Colombian regulations (Resolution 1407 of 2022). This behavior also coincides with that reported by Zhou et al. [30], who observed an increase in the number of colonies in the control treatments compared to the coated ones. In addition, the number of colonies in the control group increased significantly from day 6 onwards. Coatings with chitosan loaded with ascorbic acid and curcumin resulted in fewer colonies on the last day compared to the controls.

Regarding coliforms, no growth was observed in any of the samples, reflecting a good disinfection process. These results were consistent with those reported by [62], who evaluated an edible coating based on gelatin on strawberry quality, showing an absence of coliforms at the beginning and end of storage in strawberries coated with different concentrations and immersion times.

**Figure 6:**
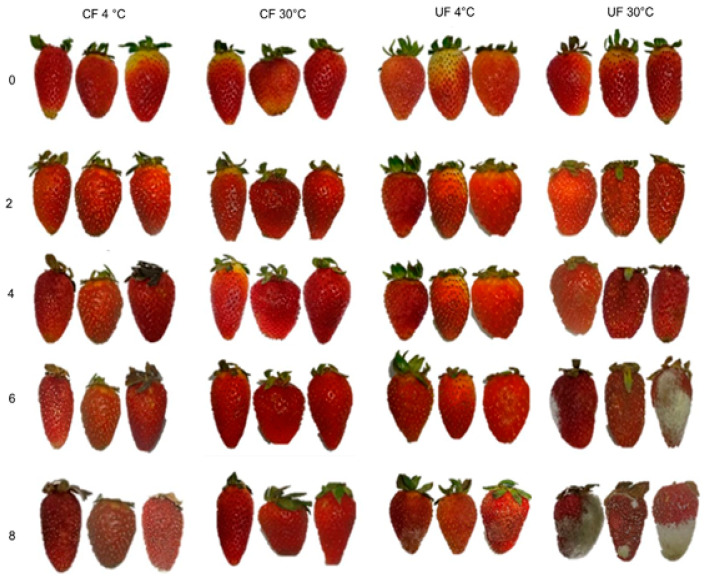
Behavior of strawberries during storage at 4 °C and 30 °C with and without edible coating.

Due to their susceptibility to tissue damage and fungal pathogens, strawberries have a very limited shelf life [63]. Although all samples initially exhibited a red color and intact structure (Day 0), storage revealed significant differences: uncoated fruits suffered the most deterioration due to enzymatic browning and mold. Particularly at 30 °C, untreated strawberries showed signs of degradation by the fourth day, reaching total decomposition by the eighth (Figure 6). In contrast, the application of the coating proved to be an effective barrier, maintaining firmness and color throughout the evaluation period. However, the application of the coating was able to preserve the color and firmness attributes of the strawberries for 8 days. Strawberries coated and stored at 4 °C showed better results in terms of quality preservation and microbiological results. The ability of the coating to maintain the fruit’s appearance for eight days is consistent with the results reported by Zhou et al. [30]. In that study, while the treated group retained its visual integrity, the uncoated samples (control) underwent significant deterioration starting on the fourth day of observation.

### 2.4. Effect of Coating on Shelf Life

The shelf-life estimation for strawberries in this study was performed using kinetic models that tracked fluctuations in weight loss and pH. These variables were selected for their critical role in assessing the fruit’s quality for consumption. According to the data in Table 3, it was established that the weight loss parameter showed zero-order reaction behavior for the coated samples at both temperatures. In contrast, a first-order reaction kinetic was observed for the uncoated samples at 30 °C. Furthermore, the zero-order model fit the coated samples at both temperatures for the pH parameter, while the first-order model was a better fit for the uncoated strawberries.

As observed in Table 4, both the application of the edible coating and refrigeration contributed to extending the shelf life of the strawberries compared to the room-temperature controls. The edible coating delayed weight loss and better stabilized the pH and soluble solids, while refrigeration acted as the decisive factor in slowing down metabolic processes. This aligns with recent studies, where the combined use of polysaccharide, chitosan, or bacterial cellulose-based coatings under cold conditions has demonstrated a relevant prolongation of strawberry shelf life, preserving sensory and nutritional quality parameters for periods of up to 15–20 days, versus less than 10 days for fruit stored at room temperature [64].

The estimated shelf life was longer than that reported by [65], who applied an edible coating based on glycerol and cinnamaldehyde, preserving strawberries for 10 days at 5 °C while maintaining firmness and overall quality. However, it was lower than that reported by [13], who used a coating with a composition similar to that used in this study with exopolysaccharide from *W. confussa* and *L. plantarum* applied to cherry tomatoes, obtaining a shelf life of 25 days at 4 °C and 11 days at 30 °C. The differences in the time determined are due to the composition of the fruit, in that strawberries are non-climacteric fruit compared to tomatoes, which are climacteric, and this fact affects the respiration rate.

### 2.5. Sensory Analysis

**Figure 7:**
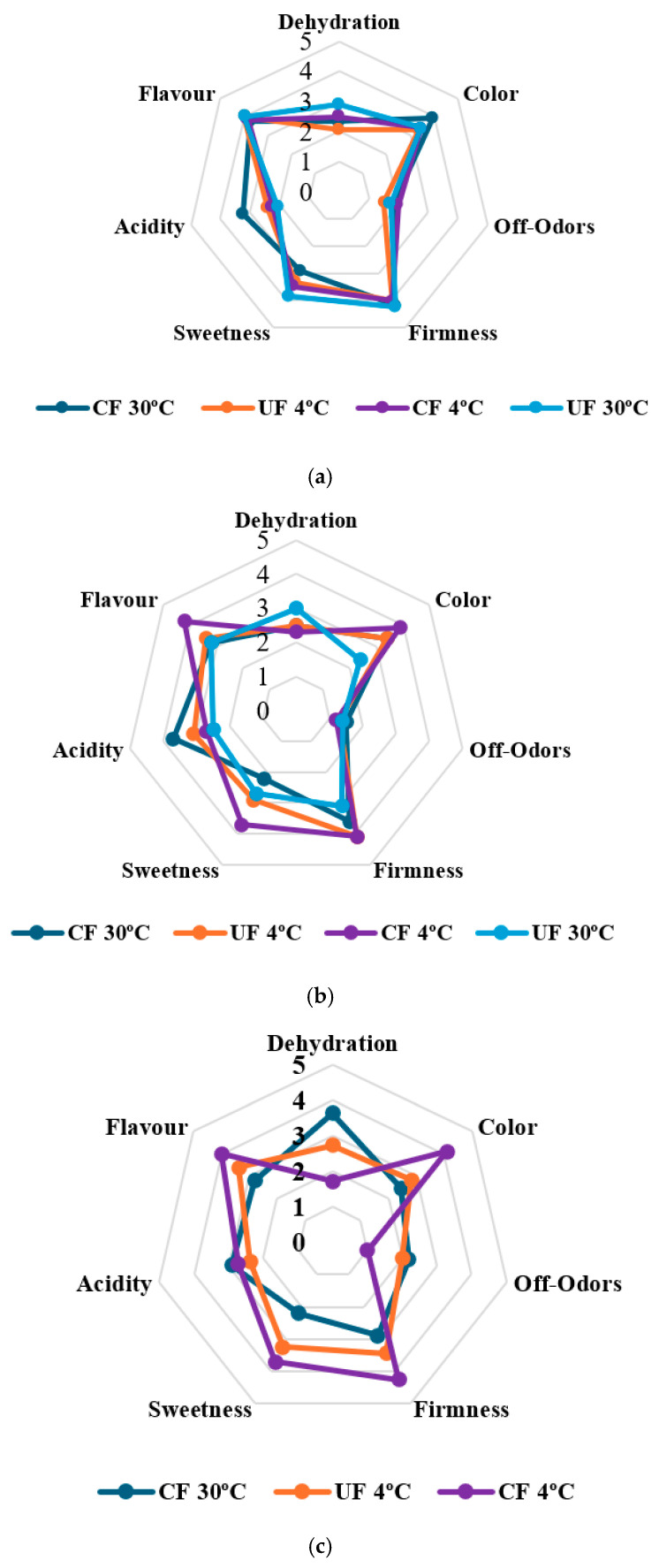
Sensory properties of coated strawberries and controls at room temperature and refrigerated for the day (**a**) 1, (**b**) 3 and 5 (**c**). Values are expressed using a hedonic scale from 1 to 5 (1 = not present/very low/atypical and 5 = very pronounced/very typical).

To verify that the addition of *L. plantarum* strains had no undesirable effects on the organoleptic quality of the fruit, sensory analysis was performed over five days. On the first day (Figure 7), the firmness attribute was very similar for all treatments, as was the flavor. However, there was a marked difference in sweetness, with the fruit coated at 30 °C being the least sweet and the most acidic, but the best rated in terms of color. This rating is in line with expectations, as it has been reported that coatings incorporating lactic acid bacteria may be associated with less sweet taste perceptions attributed to the production of organic acids such as lactic acid [66]. Applying EC improves color perception because it is associated with a lower respiration rate and ethylene production, which slows down metabolic activities in the fruit, preserving this attribute for longer [2].

Figure 7b shows that, on the third day of storage, refrigeration is the key factor in preserving the organoleptic properties of strawberries, such as firmness, color, and characteristic flavor, as well as reducing atypical odors. The edible coating, although complementary, is more effective when combined with refrigeration, helping to reduce dehydration and maintain freshness. In contrast, storage at room temperature without coating results in greater loss of quality. These results coincide with those found by [67], who pointed out that edible coatings not only act as a barrier against water loss and fungal attack, but also help retain volatile aromatic compounds, improving overall sensory acceptance. In their study, coated strawberries stored under refrigeration had a better sensory score, and coated strawberries stored at room temperature showed greater preservation of organoleptic characteristics compared to their control samples (without coating).

The analysis of variance performed shows that, for the characteristic flavor of strawberries, no significant differences (*p* > 0.05) were found between the coating and temperature conditions. This indicates that the interaction between these factors does not have a considerable effect on flavor on the third day. This result coincides with that presented by [68], where their edible coating did not affect the flavor of the coated strawberries, which means that the application of these edible coatings could be viable as they do not alter their characteristic flavor. However, for other organoleptic variables such as acidity, color, degree of dehydration, and the presence of atypical odors, the analysis of variance showed significant interactions (*p* < 0.05) between the coating and temperature. This suggests that both the type of coating and the storage temperature play important roles in modifying these properties. For example, the coating can influence color retention, keeping it more vivid and preventing strawberries from wilting at a faster rate. Van et al. [69] mention that there are certain compounds that can alter the quality of odors in coated strawberries; however, this does not affect their sensory quality and acceptability. Likewise, in terms of acidity and the presence of atypical odors, the combined effects of coating and refrigeration reduce decomposition and the production of volatile compounds, which are responsible for bad odors [67].

On the other hand, the analysis showed that the sweetness of strawberries also undergoes a significant change (*p* < 0.05), especially in uncoated strawberries. This indicates that coating has a direct impact on sweetness preservation, possibly by reducing water loss and, therefore, the concentration of the fruit’s natural sugars. The relationship between coating and temperature suggests that both factors act synergistically to maintain the sweet attributes of strawberries during storage. Gupta et al. [38] found that starch-based coatings + NADES (natural eutectic solvent) showed significant differences (*p* ≤ 0.05) between coated strawberries and the control in total soluble solids during storage at room temperature. Coated strawberries showed a delay in the decrease in soluble solids, implying better preservation of sweetness.

Finally, on day 5, the uncoated samples stored at room temperature showed signs of decomposition, making it impossible to perform the sensory analysis. On the other hand, the sample that showed greater firmness, sweetness, lower acidity, and better flavor was the coated sample stored under refrigeration, demonstrating the synergistic effect of coating and storage temperature in preserving these attributes. This is similar to what was reported by Chavan et al. [55], who state that, in general, at low storage temperatures, coatings can prevent the loss of fruit firmness. Cell turgidity, as well as the structure and composition of cell wall polysaccharides, influence the texture properties of the fruit. In addition, fruits stored at low temperatures have greater firmness and superior acceptable organoleptic quality than those stored at room temperature.

However, this sample had a lower score for dehydration because during storage at 4 °C, the relative humidity is low (50%), which promotes water exchange between the product and the environment. On the other hand, the coated strawberry at 30 °C had the highest score for this parameter, due to the higher relative humidity (77% in the city of Tuluá). This coincides with the findings of [38], who reported that the coated sample showed a lower reaction rate constant and that strawberries stored at 20 °C were able to prolong the ripening process for up to 18 days.

### 2.6. Cost–Benefit Analyses

The cost of the components to produce 300 mL of coating is shown (Table 5). The value of the exopolysaccharide was calculated considering that its production requires the preparation of an inoculum of the bacterium *Weissella confusa*. In the research by Quintero Pinilla and Florez Jaramillo [70], an approximate yield after drying of 40% of the total produced was reported, and a value of USD 1.49/g of EPS was estimated.

To estimate prices, it is important to consider the brands of each ingredient, which has an impact on the final value of edible coating. Experiments showed that 200 mL of solution is sufficient to coat approximately 1 kg of strawberries using the immersion method. Furthermore, it is suggested that future research evaluate the application of the coating by the spraying method, as this is more convenient on an industrial scale and more effective in terms of time.

The economic analysis underscores the novelty of this approach; with a production cost of 1.49 USD/g and an efficient dosage of 2.5 g per 300 mL of coating, this formulation offers a cost-effective and ‘clean label’ alternative for industrial scaling. It is important to highlight that the selection of *Weissella confusa* to produce the EPS, rather than conventional polymers such as alginate or chitosan, is justified by its unique techno-functional properties, proven efficacy in previous food models, and economic viability. Prior characterization by our research group [71] identified a protein content of 9.24 mg/L in this EPS, which significantly enhances its solubility and water-holding capacity. This protein-polysaccharide interaction creates a stable, hydrated matrix that prevents syneresis, providing a critical moisture reservoir for *L. plantarum*. The synergy between the EPS and the LAB occurs through a dual mechanism: the EPS acts as a physical ‘scaffold’ that facilitates bacterial adhesion and biofilm formation, while simultaneously protecting the cells from environmental stress. This is consistent with the studies previous findings in cherry tomatoes of our research group [13,72], where this specific EPS-LAB system ensured probiotic survival and exerted significant antifungal activity against *Fusarium* sp. and *R. stolonifer*.

## 3. Conclusions

The study showed that the EPS-based coatings effectively maintained strawberry quality during storage by delaying softening, lowering the respiration rate, and minimizing weight loss. This preservation led to a noticeable delay in physicochemical degradation. The EC preserved the microbiological quality of the strawberries, as there were no enteric microorganisms and lower CFUs in molds and yeasts compared to the control. As a result, the shelf life of the coated fruit (CF) was prolonged compared to the uncoated fruit (UF). This strongly suggests that the developed edible coating (EC) formulation is a promising technology for enhancing the postharvest preservation of strawberries. The shelf life of strawberries was estimated at up to 15 days under refrigeration (4 °C) and 6 days at room temperature (30 °C). Furthermore, sensory evaluation identified that the combination of coating and refrigeration temperature resulted in greater acceptance of the fruit in all parameters evaluated (color, flavor, firmness, sweetness, acidity, etc.).

## 4. Materials and Methods

### 4.1. Method for Generating Exopolysaccharides Through the Cultivation of W. confusa JCA4

EPS production was carried out on MRS medium, using a modified version of the method described in earlier research [73]. To begin, the JCA4 strain of *W. confusa* was cultured in MRS broth (Scharlau, Barcelona-Spain) for a day (24 h) at 37 °C. After verifying the purity of the strain, inoculation was performed on solid MRS medium using a ratio of 10 mL of liquid culture per 150 mL of solid medium, and the culture was incubated at 25 °C for 36 h. The synthesized EPS was then recovered directly from the surface of the culture medium. The sample was dried at 40 °C for 48 h. It was then pulverized using an analytical mill (IKA A11 basic, Württemberg, Germany) and filtered through a 100-mesh sieve (Fisher Scientific, Waltham, MA, USA) to ensure a particle size of less than 150 μm. To ensure the reproducibility of the experiments, the dry EPS powder was produced in several batches and stored under controlled environmental conditions (25 °C and 75% relative humidity) until it was used in the experimental replicates. The EPS (exopolysaccharide) was characterized, and prior studies confirmed the reproducibility and purity of its production process [74].

### 4.2. Fruit

Uniform strawberries (*Fragaria × ananassa* var. Chandler) standardized according to Colombian Technical Standard NTC 4103 (stage 6) [75] were selected based on the absence of physical damage or fungal signs. For surface sterilization, fruits underwent a sequential immersion protocol: first in 2% (*v*/*v*) sodium hypochlorite for 2 min, followed by 70% (*v*/*v*) ethanol for 30 s After disinfection, strawberries were rinsed with sterile water and dried at room temperature before treatment.

### 4.3. Coating Preparation and Application

Following the method of [74], the mixture was prepared under aseptic conditions inside a laminar flow hood, with the process maintained at room temperature to ensure the purity of the solution. Previously, all utensils and components were sterilized by autoclaving at 121 °C for 15 min. The composition included glycerol (Sigma-Aldrich, St. Louis, MO, USA) as a plasticizer, Tween 80 (Merck, Darmstadt, Germany) as an emulsifier, and oleic acid (Sigma-Aldrich, St. Louis, MO, USA) to enhance adhesion by acting as a surfactant. The largest proportion of the mixture was water, and this percentage remained constant. Table 6 shows the components and proportions used.

Quintero Pinilla & Florez Jaramillo [70], carried out a design of mixtures of extreme vertices of degree one, performing nine treatments. According to the results of this design, an optimal formulation was found that maximized the firmness variable and minimized weight loss. This study was carried out using this formulation (Table 7).

The edible coating was prepared mixing EPS, alginate, glycerol, and water (66% of the total), stirring it at 3500 rpm for 10 min with a MIX IMUSA 300 W mixer, and it was kept at 40 °C to facilitate the dilution of both components. This mixture was combined with Tween 80 and oleic acid by stirring for 5 min. After was then made up with the remaining water and allowed to equilibrate for 20 min (until reaching 25 °C) before proceeding with bacterial inoculation (*L. plantarum* with a viability of 8.5 CFU/mL). Then added the inoculum, the mixture was stirred for a final 2 min to ensure uniform distribution of the microorganisms throughout the matrix. The mixtures were then sealed and refrigerated at 4 °C for 24 h prior to experimental use.

Once done, it was put in 250 mL beakers where the strawberries were dipped one by one to make sure they were totally covered for 5 min. To remove excess liquid, the samples were transferred to stainless steel sieves and then dried under laminar flow conditions at 25 °C until a uniform layer was obtained. The treated strawberries were placed in transparent PET containers. The samples were then stored for 8 days under two temperature conditions: 4 °C and 30 °C. The storage at 4 °C and 30 °C was maintained using a Peltier-cooled incubator, model IPP110eco (Memmert, Schwabach-Germany). Finally, the strawberries were removed from both environments to determine their physicochemical indicators at 0, 2, 4, 6, and 8 days of storage. A figure is presented illustrating the procedure applied for the development and application of the coating (Figure S1. Methodology for the development and evaluation of strawberry coatings during storage). 

### 4.4. Experimental Design

A completely randomized factorial design (2 × 2) was used to conduct this study. Details are presented in Table 8.

The strawberries were stored at 30 °C as a post-harvest thermal stress condition, since high temperatures increase the respiratory rate, accelerate physiological deterioration, and promote losses in fruit quality and weight, thus allowing the effectiveness of the coating to be evaluated. On the other hand, the refrigeration temperature of 4 °C was used as the optimal storage condition typical for highly perishable fruits, because cold storage significantly reduces the respiration and metabolic activity of the fruit, delaying quality degradation, prolonging firmness, and decreasing moisture loss and microbiological deterioration compared to higher temperatures [76].

The physicochemical tests were performed in triplicate every two days and microbiological analyses in triplicate on days 0 and 8. Respiration and gas analysis was performed using Felix F-950 Three Gas Analyzer (Felix Instruments, Camas, WA, USA) equipment every two days for 6 days, and finally, sensory analysis was performed on days 1, 3 and 5 by a panel of twelve trained judges using a 5-point hedonic scale.

### 4.5. Effect of Coating on Strawberry Physicochemical Quality

The evaluation of the shelf life and physicochemical parameters of the treated strawberries was based on a two-factor experimental design, with the application of the edible coating (EC) and storage temperature conditions as the main variables. For the analysis of the coating factor, two experimental conditions were defined: coated fruit (CF) and uncoated fruit (UF). Similarly, the temperature factor encompassed two storage conditions: controlled refrigeration (4 °C) and room temperature (30 °C). The storage at 4 °C and 30 °C was maintained using a Peltier-cooled incubator, model IPP110eco (Memmert, Schwabach, Germany). In order to analyze the effects of each treatment at specific time points, the storage period (days) was used as a blocking factor in the statistical model. Each treatment had three experimental units and was performed in triplicate. For physicochemical analysis, samples were collected every two days for up to eight days, or until the fruit quality degraded beyond the point of analysis, at both 4 °C and 30 °C.

#### 4.5.1. Weight Loss

To determine weight loss, the percentage difference between the final and initial weight of each sample was calculated. A BAS 31 weighing scale (BOECO, Hamburg, Germany), was utilized. The precise formula for calculating the percentage of weight loss in the strawberries is presented in Equation (1).

(1) $$Weight\;loss\;\left(\%\right)=\frac{W_{i}-W_{f}}{W_{f}}\times 100$$

$W_{i}$: strawberry weight at 0 d

$W_{f}$: strawberry weight at storage time t.

#### 4.5.2. Firmness

Fruit firmness was determined using a TX-700 texture analyzer (Lamy Rheology, Champagne-au-Mont-d’Or, France) equipped with a 50 N load cell. The evaluation was performed by measuring the maximum force (N) required to insert a 3 mm diameter blunt metal plunger into the equatorial zone of the strawberry. The instrument was set to a penetration depth of 10 mm at a constant test speed of 50 mm/min. For each experimental treatment, three replicates were measured to ensure statistical consistency.

#### 4.5.3. Color

The surface color of the strawberries was characterized using a NH310 colorimeter (3NH Technology Co., Ltd., Shenzhen, China). The device was operated with a D65 illuminant and a 10° standard observer, using an 8 mm aperture. Before measurements, the instrument was calibrated with standards. Color coordinates were recorded in the CIELab scale (L*, a*, and b*) at three equidistant points along the equatorial section of each fruit. Additionally, the Hue angle (Hue) (Equation (2)) was calculated to evaluate the evolution of the fruit characteristic redness during storage.

(2) $$Hue={tan}^{-1}\left(\frac{b^{*}}{a^{*}}\right)$$

where

a*: Green-Red Coordinate

b*: Blue-Yellow Coordinate.

#### 4.5.4. Internal Fruit Quality

To prepare the samples, strawberries were macerated for two minutes using a MiniMix homogenizer 100WCC (Interscience, Saint-Nom-la-Bretèche, France), and the resulting fruit juice was collected for analysis. The titratable acidity (TA) of the juice was measured via the AOAC 942.15 method. This involved titrating 5 g of filtered juice (diluted in 100 mL of deionized water) with a 0.1 mol/L NaOH solution until a pH of 8.1 was reached, using phenolphthalein as the indicator. The results, expressed as percent citric acid, were calculated using Equation (3):

(3) $$Titratable\;acidity\;(\%)=\frac{V_{NaOH}\;\times \;0.1\;\times \;0.064}{V_{m}}\times \;100$$

The pH of the samples was determined following the AOAC 981.12 method using an Orion 3 Star pH-meter (Thermo Scientific, Waltham, MA, USA). Total Soluble Solids (TSS) were measured with a digital refractometer (RX-700, Atago Co., Ltd., Tokyo, Japan) (accuracy ±0.1%) according to the AOAC 932.12 method (AOAC, 2016). All analyses for both storage conditions were conducted with three replicates per treatment (n = 3).

### 4.6. Production of CO_2_ and Ethylene (C_2_H_4_) and Consumption of O_2_

Gas exchange measurements compared the CF to the UF control under simulated tropical market conditions: storage at 30 °C and 75% RH. These measurements were repeated every two days for eight days. CO_2_ and C_2_H_4_ production and O_2_ consumption were tracked in a respiration chamber for 60 min using a portable gas analyzer (F-950 Three Gas Analyzer; Felix Instruments, Camas, WA, USA). Concentrations were determined using the specific equations outlined by Salas-Mendez et al. [77].

(4) $$\frac{{dy}_{O_{2}}}{dt}=-X_{O_{2}}\frac{W_{t}}{V_{f}}$$

(5) $$\frac{{dy}_{{CO}_{2}}}{dt}=-X_{{CO}_{2}}\frac{W_{t}}{V_{f}}$$

(6) $$\frac{{dy}_{C_{2H_{4}}}}{dt}=-X_{C_{2}H_{4}}\frac{W_{t}}{V_{f}}$$

where $X_{O_{2}}$, $X_{{CO}_{2}}$ and $X_{C_{2}H_{4}}$ represent oxygen consumption

### 4.7. Microbiological Analysis

The microbiological quality assessment was performed on days 0 and 8 for all samples stored at both 4 °C and 30 °C. This methodology was based on the approach by Alvarez et al. [13], incorporating specific modifications. To prepare the samples for plate counting, the strawberries were first homogenized and then serially diluted (10^−1^ to 10^−6^) in peptone water. Quality was assessed using indicator microorganisms, including mesophiles, total enterics, total and fecal coliforms, and yeasts and molds. Specifically, yeasts and molds were quantified using Potato Dextrose Agar (PDA, Condolab, Spain), mesophiles on Plate Count Agar (PCA, Condolab, Spain), total enterics on Violet Red Bile Glucose Agar (VRBG, Condolab, Spain). A 100 μL aliquot of the prepared solution was inoculated onto each Petri dish. Plates for mesophiles and enterics were incubated at 35 °C for 48 h, while those for molds and yeasts were incubated at 25 °C for 5 days before colony counting, with results reported as CFU of fruit. Furthermore, total and fecal coliforms were quantified using the Most Probable Number (MPN) method in sets of three tubes. The culture medium used was Lauryl Sulfate Broth (Scharlau, Barcelona, Spain), incubated at 35 °C for 24 h. It should be noted that each count was performed in three independent replicates (n = 3).

### 4.8. Analysis of Strawberry Shelf Life

The shelf life of the coated fruit (CF) and uncoated fruit (UF) experimental units was evaluated over an 8-day period under two distinct storage temperatures: 4 °C and 30 °C. To calculate the shelf life, the first step was determined the reaction order for each physicochemical parameter. Consistent with established literature, the shelf life was specifically defined as the point in time when any of the monitored physicochemical parameters reached its pre-determined maximum or minimum threshold value [78].

(7) $$\frac{d\left[A\right]}{dt}=k{\left[A\right]}^{n}$$

*A*: Concentration or value of the parameter to be evaluated

*n*: Reaction order

*k*: Reaction constant (dependent on n)

*t*: Time (d)

The kinetics of each attribute were established by determining its reaction order using Equation (8). The integrated versions of this model, which cover the most frequently encountered reactions in food products—specifically zero-order (Equation (8)), first-order (Equation (9)), and second-order (Equation (10))—were utilized for analysis [78,79]. To identify the most accurate kinetic representation, the best-fit model for each parameter was selected based on the maximum correlation coefficient (R^2^). This process was carried out individually for each treatment (CF or UF) under the different storage temperatures.

(8) $${\left[A\right]}_{t}=A_{0}-K_{c}t;\;n=0$$

(9) $${\left[A\right]}_{t}=A_{0}e^{-k_{c}t};n=1$$

(10) $$\frac{1}{{\left[A\right]}_{t}}=K_{c}t+\frac{1}{A_{0}};n=2$$

Using the chosen best-fit models, we determined the reaction constant (*k*) and the initial value (*A_0_*) for each parameter. These calculated values were then utilized to establish the shelf life of the product. The threshold values defined for each parameter were:

Weight loss: maximum of 6% [80]

pH: maximum of 3.9 [52].

### 4.9. Sensory Evaluation

A sensory evaluation was carried out following the methodology described by De Simone et al. [69]. Coated strawberries from days 1, 3 and 5 of storage were evaluated using a panel of twelve previously trained judges. The training phase involved familiarization and calibration of the panelists for the identification and scoring of key sensory attributes associated with fruit quality, such as translucency, degree of dehydration, firmness, juiciness, sweetness, characteristic flavor, presence of off-odors, and color.

The evaluation was performed using a 5-point hedonic scale, where 1 represented a very low intensity or absence of the attribute, and 5 corresponded to a high intensity or a typical characteristic of the fresh fruit.

## Figures and Tables

**Table 1:** Color parameter results for coated and uncoated treatments.

Storage Time (d)	30 °C		4 °C	
	UF	CF	UF	CF
L*				
0	38.70 ± 3.82 ^NS^	39.40 ± 3.58 ^NS^	37.73 ± 0.51 ^NS^	39.77 ± 3.52 ^NS^
2	32.29 ± 0.65 ^b^	36.37 ± 1.03 ^ab^	22.28 ± 2.43 ^c^	37.43 ± 2.36 ^a^
4	25.14 ± 2.28 ^b^	33.46 ± 0.79 ^a^	18.31 ± 2.57 ^c^	34.16 ± 1.82 ^a^
6	11.14 ± 1.55 ^b^	16.24 ± 0.95 ^b^	15.92 ± 2.88 ^b^	28.16 ± 3.99 ^a^
8	8.92 ± 1.13 ^b^	13.88 ± 0.12 ^b^	11.99 ± 1.76 ^b^	21.22 ± 2.77 ^a^
a*				
0	29.63 ± 2.50 ^a^	31.27 ± 0.97 ^a^	37.39 ± 0.47 ^b^	38.53 ± 0.95 ^b^
2	34.30 ± 0.34 ^a^	33.12 ± 0.72 ^a^	37.73 ± 0.81 ^b^	37.48 ± 1.19 ^b^
4	41.74 ± 0.44 ^b^	33.91 ± 0.85 ^d^	49.59 ± 0.58 ^a^	39.19 ± 1.20 ^c^
6	26.17 ± 3.21 ^a^	27.77 ± 1.81 ^a^	40.97 ± 0.65 ^b^	38.82 ± 0.69 ^b^
8	21.21 ± 0.32 ^c^	26.57 ± 0.58 ^b^	37.33 ± 0.45 ^a^	38.31 ± 0.38 ^a^
b*				
0	35.17 ± 3.65 ^b^	42.24 ± 3.28 ^a^	39.62 ± 0.57 ^ab^	38.39 ± 0.21 ^ab^
2	34.55 ± 0.50 ^a^	38.84 ± 1.97 ^b^	36.48 ± 2.18 ^a^	37.87 ± 0.57 ^b^
4	27.17 ± 3.22 ^c^	33.13 ± 0.66 ^ab^	30.85 ± 0.56 ^bc^	36.28 ± 0.35 ^a^
6	21.97 ± 0.43 ^c^	23.61 ± 0.71 ^bc^	27.65 ± 3.03 ^b^	32.71 ± 0.26 ^a^
8	17.96 ± 0.80 ^c^	22.85 ± 0.24 ^b^	18.85 ± 1.38 ^c^	29.35 ± 2.02 ^a^
H*				
0	49.85 ± 1.98 ^ab^	53.43 ± 1.71 ^a^	46.66 ± 0.10 ^b^	46.43 ± 0.89 ^b^
2	45.20 ± 0.52 ^a^	46.52 ± 2.05 ^a^	44.00 ± 1.17 ^b^	44.98 ± 0.44 ^b^
4	32.98 ± 3.00 ^a^	44.33 ± 1.25 ^b^	31.88 ± 0.68 ^a^	42.80 ± 0.88 ^b^
6	40.18 ± 3.53 ^a^	40.42 ± 2.13 ^a^	33.95 ± 2.99 ^a^	40.12 ± 0.66 ^a^
8	40.23 ± 1.27 ^a^	40.69 ± 0.84 ^a^	27.43 ± 1.39 ^b^	26.77 ± 1.92 ^b^

Data are means ± SD (n = 3). Different letters in each column show significant difference (*p* < 0.05). NS: Not significant.

**Table 2:** Effect of Coating Application on the Total Soluble Solids, Titratable Acidity and pH of Strawberries (CF and UF) during Storage at 4 °C and 30 °C.

Time (d)	30 °C		4 °C	
	UF	CF	UF	CF
	pH			
0	3.35 ± 0.05 ^a^	3.45 ± 0.05 ^a^	3.41 ± 0.05 ^a^	3.48 ± 0.05 ^a^
2	3.55 ± 0.01 ^b^	3.45 ± 0.01 ^c^	3.40 ± 0.05 ^a^	3.47 ± 0.01 ^c^
4	3.55 ± 0.01 ^a^	3.49 ± 0.005 ^a^	3.57 ± 0.07 ^a^	3.38 ± 0.01 ^b^
6	3.70 ± 0.01 ^a^	3.5 ± 0.01 ^b^	3.43 ± 0.01 ^c^	3.48 ± 0.005 ^d^
8	3.60 ± 0.02 ^a^	3.58 ± 0.005 ^ab^	3.55 ± 0.02 ^ab^	3.52 ± 0.01 ^b^
	Titratable Acidity (% citric acid)			
0	1.10 ± 0.04 ^a^	1.10 ± 0.04 ^a^	1.10 ± 0.04 ^a^	1.10 ± 0.04 ^a^
2	1.00 ± 0.01 ^ab^	1.11 ± 0.04 ^a^	1.13 ± 0.004 ^b^	1.00 ± 0.01 ^ab^
4	0.91 ± 0.01 ^b^	1.09 ± 0.02 ^a^	0.95 ± 0.03 ^a^	0.98 ± 0.02 ^b^
6	0.89 ± 0.01 ^a^	1.04 ± 0.03 ^a^	0.86 ± 0.03 ^a^	1.00 ± 0.02 ^b^
8	0.80 ± 0.01 ^b^	0.88 ± 0.02 ^c^	0.71 ± 0.02 ^a^	0.93 ± 0.02 ^a^
	TSS (%)			
0	9.26 ± 0.05 ^a^	9.26 ± 0.05 ^a^	9.26 ± 0.05 ^a^	9.26 ± 0.05 ^a^
2	8.90 ± 0.10 ^b^	8.23 ± 0.06 ^c^	9.97 ± 0.15 ^a^	8.17 ± 0.06 ^c^
4	7.90 ± 0.10 ^b^	9.10 ± 0.10 ^a^	9.27 ± 0.15 ^a^	8.07 ± 0.25 ^b^
6	7.60 ± 0.10 ^a^	8.50 ± 0.0 ^b^	9.33 ±0.15 ^c^	8.13 ± 0.05 ^d^
8	8.13 ± 0.12 ^ab^	7.83 ± 0.05 ^b^	8.40 ± 0.2 ^a^	8.47± 0.11 ^a^

Values with different superscript letters (a–d) on the same bar indicate significant differences (*p* < 0.05).

**Table 3:** Coefficients of determination (R^2^) for the zero-, first-, and second-order reactions, at temperatures of 4 °C and 30 °C for coated (CF) and uncoated (UF) strawberry fruit.

Attribute [A]t	T (°C)	n = 0	n = 1	n = 2
CF				
Weight loss	4	0.9748	0.927	0.9616
	30	0.9675	0.9574	0.9091
pH	4	0.608	0.6067	0.5041
	30	0.9297	0.9266	0.8789
UF				
Weight loss	4	0.948	0.9447	0.9096
	30	0.9441	0.9614	0.9105
pH	4	0.5106	0.5161	0.5014
	30	0.6692	0.670	0.5208

**Table 4:** Shelf life of strawberries according to the degradation kinetics models, values in days.

Treatment	4 °C		30 °C	
	Weight Loss	pH	Weight Loss	pH
CF	15	15	6	10
UF	10	12	6	8

**Table 5:** Price in dollars/g of edible coating.

Materials	Quantity (g)	Price/g	Cost (USD)
Sodium alginate	0.08	0.05	0.004
EPS	2.5	1.49	3.725
Materials	Quantity (mL)	Price/mL	Cost (USD)
Water	189	0.01	1.89
Glycerol	3.42	0.08	0.27
Oleic acid	2	0.07	0.14
Inoculum (*L. plantarum*)	2	0.21	0.42
Tween 80	1	0.02	0.02
Total cost (300 mL)			6.50

**Table 6:** Compounds and proportions for coating production.

Compound	Quantity (%)
Water	94.5
EPS	3 *
Sodium alginate	
Glycerol	
Oleic acid	1
*L. plantarum*	1
Tween 80	0.5

* EPS, sodium alginate, and glycerol combined must not account for more than 3% of the total formulation.

**Table 7:** Optimized coating formulation.

Sodium Alginate (%)	EPS (%)	Glycerol (%)
0.04	1.25	1.71

**Table 8:** Experimental matrix and analyzed parameters.

Factor	Levels
Type of treatment	Uncoated strawberry (Control)
	Strawberry with optimized edible coating
Storage temperature	Refrigeration (4 °C)
	Controlled ambient conditions (30 °C)
Evaluation	Physicochemical every two days (0, 2, 4, 6, 8 days) microbiological on days 0 and 8.

## Data Availability

The data used to support the findings of this study can be made available by the corresponding author upon request.

## Supplementary Materials

The following supporting information can be downloaded at: https://www.mdpi.com/article/10.3390/gels12040341/s1, Figure S1: Methodology for the development and evaluation of strawberry coatings during storage.
