# Supplementary material for: Efficacy of EPS Gel Coating and Lactic Acid Bacteria in Preserving Strawberry Postharvest Quality
Source: Gels. 2026 Apr 19;12(4):341. doi: 10.3390/gels12040341 (PMC13115376; doi:10.3390/gels12040341)
Supplement: Supplementary file 1 [file gels-12-00341-s001.zip › gels-4237628-supplementary.pdf]

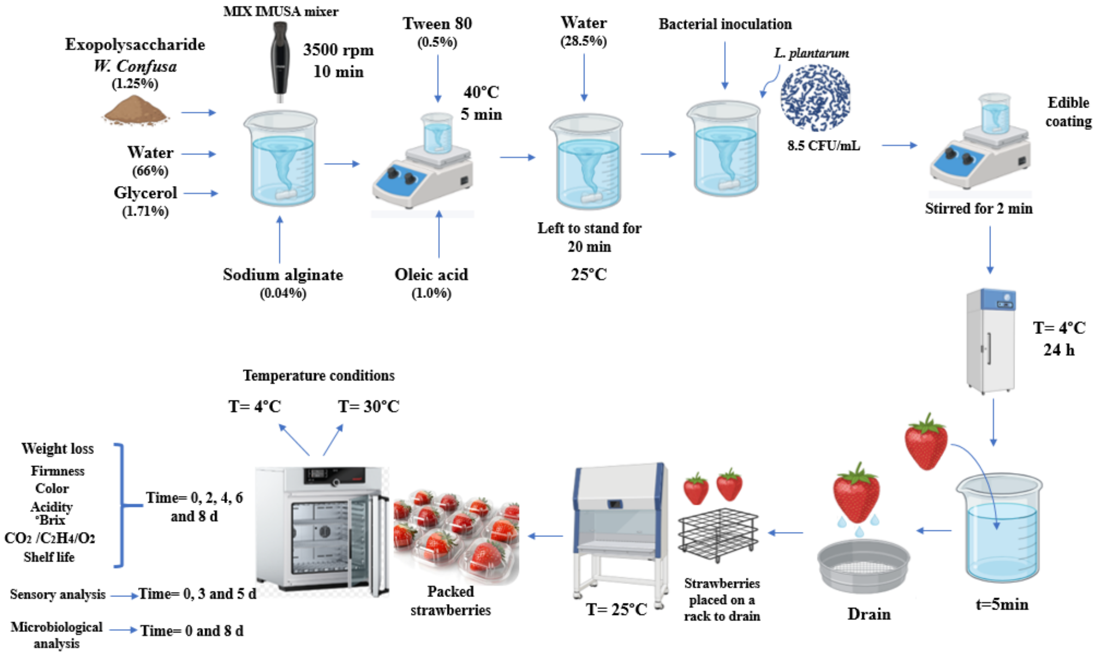

Figure S1. Methodology for the development and evaluation of strawberry coatings during storage
